# Prognostic Significance of Preoperative Albumin-Globulin Ratio in Patients with Upper Tract Urothelial Carcinoma

**DOI:** 10.1371/journal.pone.0144961

**Published:** 2015-12-17

**Authors:** Bo Zhang, Wei Yu, Li-Qun Zhou, Zhi-Song He, Cheng Shen, Qun He, Jun Li, Li-Bo Liu, Cong Wang, Xiao-Yu Chen, Yu Fan, Shuai Hu, Lei Zhang, Wen-Ke Han, Jie Jin

**Affiliations:** 1 Department of Urology, Peking University First Hospital, Beijing, People’s Republic of China; 2 Institute of Urology, Peking University, Beijing, People’s Republic of China; 3 National Urological Cancer Center, Beijing, People’s Republic of China; 4 Peking University Health Science Center, Beijing, People’s Republic of China; Sun Yat-sen University, CHINA

## Abstract

**Background:**

Preoperative albumin-globulin ratio (AGR) reflects both malnutrition and systemic inflammation in cancer patients. In particular, systemic inflammation has been reported to contribute to tumor progression and poor oncological outcome in various malignancies. However, the prognostic value of preoperative AGR in upper tract urothelial carcinoma (UTUC) has not been examined.

**Methods:**

We retrospectively reviewed medical data of 187 operable UTUC patients in a Chinese cohort with a high incidence of chronic kidney disease (CKD). AGR was calculated as [AGR = albumin/(serum total protein—albumin)]. The associations of preoperative AGR with clinicopathologic characteristics and prognosis were assessed. Multivariate analyses using Cox regression models were performed to determine the independent prognostic factors.

**Results:**

The median (IQR) preoperative AGR was 1.50 (1.30–1.70), and the optimal cutoff value was determined to be 1.45 according to the receiver operating curve analysis. Low AGR was significantly associated with female gender, high CKD stage and tumor grade (*P* < 0.05). Eighty-three patients died before the follow-up endpoint. Kaplan-Meier analysis showed that an AGR < 1.45 predicted significantly poorer overall and cancer-specific survivals compared to an AGR ≥ 1.45 (*P* < 0.001 and *P* = 0.008, respectively). Multivariate analyses showed that an AGR < 1.45 was an independent risk factor for poorer overall and cancer-specific survivals (*P* = 0.002 and *P* = 0.015, respectively).

**Conclusions:**

Preoperative AGR can act as an effective biomarker with easy accessibility for evaluating the prognosis of patients with UTUC. AGR should be applied in UTUC patients for risk stratification and determination of optimal therapeutic regimens.

## Introduction

Upper tract urothelial carcinoma (UTUC) is a rare malignancy in western countries, comprising merely ≈5% of urothelial tumors [1, 2]. In contrast, it is more common in the Chinese population for several reasons, particularly the consumption of Chinese herbs containing aristolochic acid (AA) [1]. Prior studies in Taiwan have reported that UTUC accounts for 31% of all urinary tract urothelial carcinomas and is often complicated by chronic kidney disease (CKD) [3, 4]. Renal function in these patients becomes further impaired after radical nephroureterectomy (RNU). However, some patients with UTUC die from recurrence and distant metastasis even after RNU, which indicates that adjuvant therapy should be considered for this high-risk subset. In contrast, patients with low-risk disease may benefit from a more conservative treatment approach [5]. Recently, multiple postoperative parameters have been proposed for predicting prognosis, such as lymphovascular invasion, concomitant carcinoma in situ, tumor necrosis, and Snail and E-cadherin levels [5, 6]. Because of the decrease in renal function after RNU, neoadjuvant chemotherapy has been more frequently recommended than postoperative cisplatin-based chemotherapy in recent years, and this highlights the importance of simple and effective preoperative prognostic predictors.

Malnutrition and systemic inflammation promote tumor progression by destroying immune function and altering biological features of tumor cells, which results in poor oncological outcomes [7, 8]. Albumin and globulin are two major constituents of human serum total protein. Albumin not only reflects patient nutritional status but is also associated with systemic inflammation [9]. Hypoalbuminemia has been reported to be an independent risk factor of poor survival in UTUC patients [10]. On the other hand, globulin plays an important role in immunity and inflammation and serves as a carrier of sex hormones. Previous studies identified the preoperative albumin to globulin ratio (AGR) as a simple and useful predictive biomarker for evaluation of prognosis in several cancers [11–14]. Therefore, we speculate that preoperative AGR impacts the clinical outcomes of UTUC. To date, the prognostic value of AGR in UTUC has not been discussed. In the present study, we evaluated the prognostic value of preoperative AGR for predicting survival in patients with UTUC.

## Materials and Methods

### Study design and population

We retrospectively reviewed clinical data for 211 patients who were diagnosed with UTUC and received RNU from January 2006 to December 2008 at our center. To clarify, a pending related manuscript using the same cohort of patients was also submitted to PLOS ONE (PONE-D-15-26450). All patients underwent cystoscopy, ultrasound, computer tomography/magnetic resonance imaging, and/or ureteroscopy with tissue biopsy before RNU. The exclusion criteria were as follows: patients without complete laboratory test information, especially AGR; those with liver disease, autoimmune disease or multiple myeloma; those treated with conservative surgery instead of RNU; and those who received preoperative chemotherapy. Overall, 187 patients with complete follow-up information were included in the analyses. The data of patient demographic features, past and personal histories, clinicopathologic characteristics, laboratory test results, treatment methods and survival status were collected from a database containing comprehensive medical records for UTUC patients. The tumors were staged based on the 2002 Union for International Cancer Control (UICC) TNM classification system. Tumor grading was assessed according to the World Health Organization (WHO) 2004 grading system. AGR was calculated as AGR = albumin/(total protein—albumin). The glomerular filtration rate (GFR) was calculated using the CKD-EPI equation [15]. CKD stages were classified according to GFR (mL/min/1.73 m^2^) measured before surgery for UTUC as stage 5 (GFR <15 or renal replacement therapy), stage 4 (GFR: 15–29), stage 3 (GFR: 30–59), stage 2 (GFR: 60–89) or stage 1 (GFR ≥90). Patients were followed every 3 months for the first 2 years after RNU and annually thereafter. The follow-up assessments included routine blood and serum chemistry studies, cystoscopy, chest x-rays, and ultrasound/computed tomography/magnetic resonance imaging. The co-primary endpoints of the study were all-cause and cancer-specific deaths. Overall survival (OS), cancer-specific survival (CSS), intravesical recurrence-free survival (IRFS) and contralateral recurrence-free survival (CRFS) periods were calculated from the date of RNU to the date of all-cause death, cancer-specific death, and intravesical and contralateral recurrence. The causes of death were determined by reviewing death certificates or by the treating clinician. Intravesical and contralateral recurrences were diagnosed by postoperative pathology. This study was approved by the institutional ethics committee of Peking University First Hospital. As a retrospective analysis of routine data, a waiver of written informed consent was granted from the ethics committee. Patient records/information was anonymized and de-identified prior to analysis.

### Statistical analysis

Continuous variables are presented as median (IQR) and were comparatively analyzed using the independent-samples *t* test or Mann-Whitney *U* test. Categorical variables are presented as frequencies and percentages and were analyzed using the chi-squared test or Mann-Whitney *U* test. The associations of AGR with inflammatory markers (including leukocyte, neutrophil and platelet counts) and GFR were examined by correlation analysis. OS, CSS, IRFS and CRFS were analyzed using the Kaplan-Meier method and log-rank test. A univariate analysis was performed for each variable that may predict mortality using a Cox regression model. Then, the statistically and clinically significant variables were analyzed using a multivariate Cox regression model. If continuous variables were statistically significant, they were further analyzed as binary variables. Albumin and serum total protein were excluded, as they were used in the calculation of AGR. The optimal cutoff value for AGR was determined by applying receiver operating curve (ROC) analysis, as previously described [16]. The threshold that best discriminated between survival and all-cause death (in terms of sensitivity and specificity) was used, allowing us to treat AGR as a dichotomous variable. All statistical analyses were performed using the IBM Statistical Package for Social Sciences (SPSS) version 20.0. A result was considered statistically significant with a *P* value of <0.05.

## Results

### Characteristics of the entire cohort

**Fig 1:**
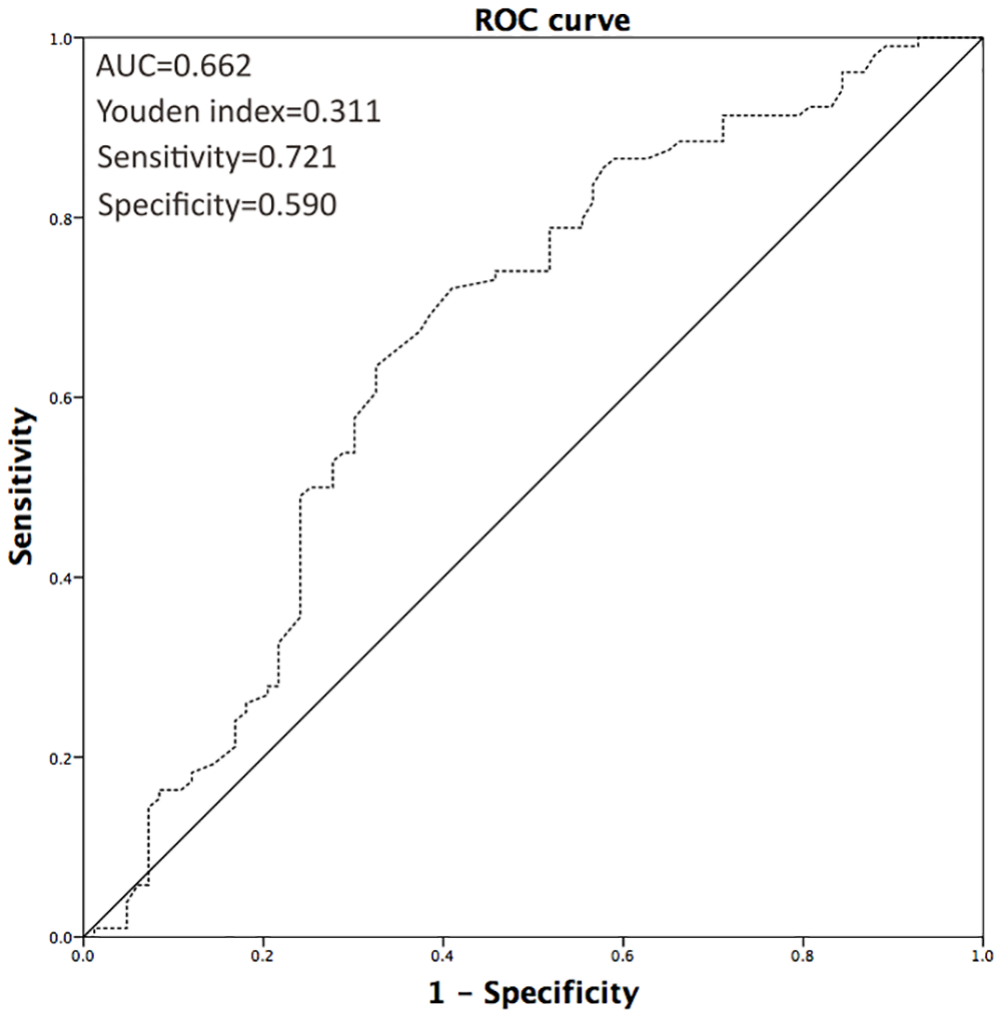
ROC analysis of optimal AGR cutoff.

Demographic and clinicopathologic characteristics of the 187 patients, including 85 (45.5%) males and 102 (54.5%) females, are summarized in Table 1. The median (IQR) age of all patients was 70 (61–74) years, and the median follow-up duration was 78 (32–92) months. The median preoperative AGR was 1.50 (1.30–1.70), and an optimal cutoff of 1.45 was determined according to the ROC analysis (Fig 1). Based on the 2002 UICC TNM classification system, 147 (78.6%) patients had localized disease (pTa-2), 40 (21.4%) had advanced disease (pT3-4), and 8 (4.3%) were pathologically proven to be N_+_; furthermore, no distant metastases were found at initial diagnosis for any of the study subjects. According to the WHO 2004 grading system, 42 (22.5%) and 145 (77.5%) cases were diagnosed as low and high grade, respectively.

**Table 1:** Baseline and clinicopathologic characteristics of 187 patients with UTUC grouped by AGR.

Variables	Low AGR group (AGR<1.45)	High AGR group (AGR≥1.45)	P value
Number of patients	78	109	
Age, year	72 (65–76)	70 (59–74)	0.028
Follow-up, months	63 (24–91)	84 (61–92)	0.006
Gender, female, n (%)	53 (67.9)	49 (45.0)	0.002
Body mass index, kg/m^2^	24.33 (21.95–27.02)	23.46 (21.47–26.40)	0.280
CHEH, n (%)	15 (19.2)	14 (12.8)	0.234
Preoperative CKD stage			<0.001
No CKD/Stage 1/Stage 2	17 (21.8)	58 (53.2)	
Stage 3	41 (52.6)	38 (34.9)	
Stage 4/Stage 5	20 (25.6)	13 (11.9)	
Preoperative GFR	48.64 (31.87–58.92)	61.40 (45.11–76.60)	<0.001
Smoking history, n (%)	9 (11.5)	21 (19.3)	0.156
Previous or synchronous BUC, n (%)	14 (17.9)	15 (13.8)	0.435
ASA score ≥III, n (%)	28 (35.9)	18 (16.5)	0.002
Hydronephrosis, n (%)	38 (48.7)	36 (38.7)	0.031
Surgical procedure, open, n (%)	56 (71.8)	71 (65.1)	0.336
Ureter involvement, n (%)			0.219
Absent	38 (48.7)	63 (57.8)	
Present	40 (51.3)	46 (42.2)	
Multifocality, n (%)	17 (21.8)	30 (27.5)	0.373
Tumor architecture, n (%)			0.075
Papillary	63 (80.8)	98 (89.9)	
Sessile	15 (19.2)	11 (10.1)	
Pathological T stage, n (%)			0.119
Localized (pTa-2)	57 (73.1)	90 (82.6)	
Advanced (pT3-4)	21 (26.9)	19 (17.4)	
Lymph node status, n (%)			0.394
N_0_ or N_x_	73 (93.6)	106 (97.2)	
N_+_	5 (6.4)	3 (2.8)	
Tumor grade, n (%)			<0.001
Low	5 (6.4)	37 (33.9)	
High	73 (93.6)	72 (66.1)	
Lymphovascular invasion, n (%)	13 (16.7)	15 (13.8)	0.583
Serum albumin, g/L	38.7 (36.5–42.0)	41.6 (39.3–43.7)	<0.001

UTUC, upper tract urothelial carcinoma; AGR, albumin-globulin ratio; CHEH, Chinese herbs exposure history; CKD, chronic kidney disease; BUC, bladder urothelial carcinoma; ASA, American Society of Anesthesiologists.

### Association of preoperative AGR with clinical characteristics

**Fig 2:**
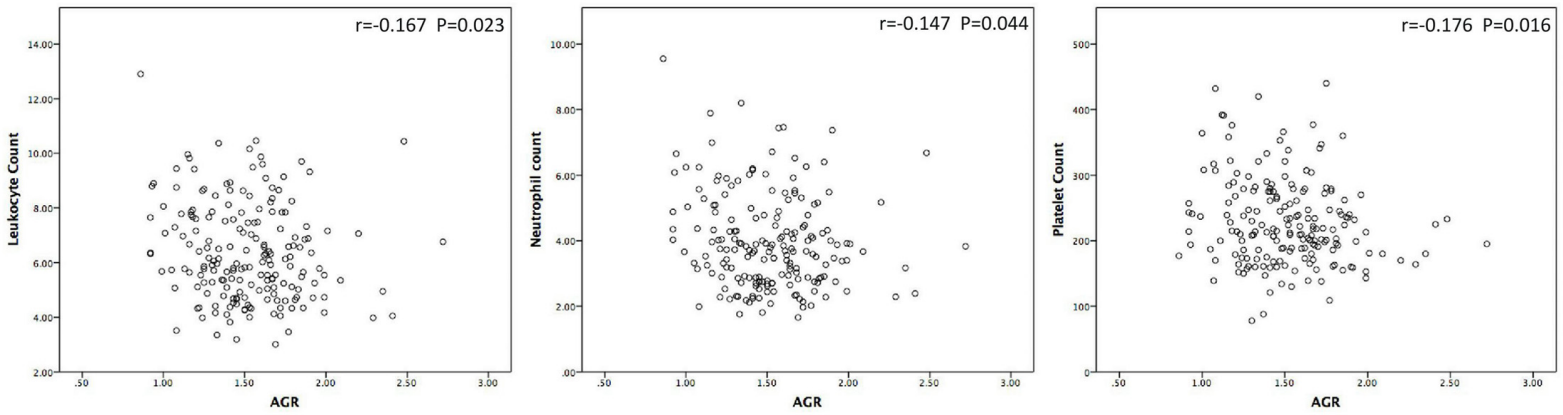
Correlation between preoperative AGR and inflammatory parameters (namely leukocyte, neutrophil and platelet counts).

This cohort was divided into two groups using the AGR cutoff of 1.45. There were no significant differences between the two groups in terms of smoking history, previous or synchronous BUC, surgical procedure, multifocality, tumor architecture, pathological T stage or lymph node status (*P* > 0.05). However, the low AGR group was significantly associated with old age (*P* = 0.028), female gender (*P* = 0.002), hydronephrosis (*P* = 0.031), low preoperative GFR (*P* < 0.001), and high preoperative CKD stage (*P* < 0.001), ASA score (*P* = 0.002) and tumor grade (*P* < 0.001). Additionally, the correlation analysis showed that AGR was negatively correlated with leukocyte, neutrophil and platelet counts (*P* = 0.023, *P* = 0.044 and *P* = 0.016, respectively) (Fig 2).

### Association of preoperative AGR with survival

**Fig 3:**
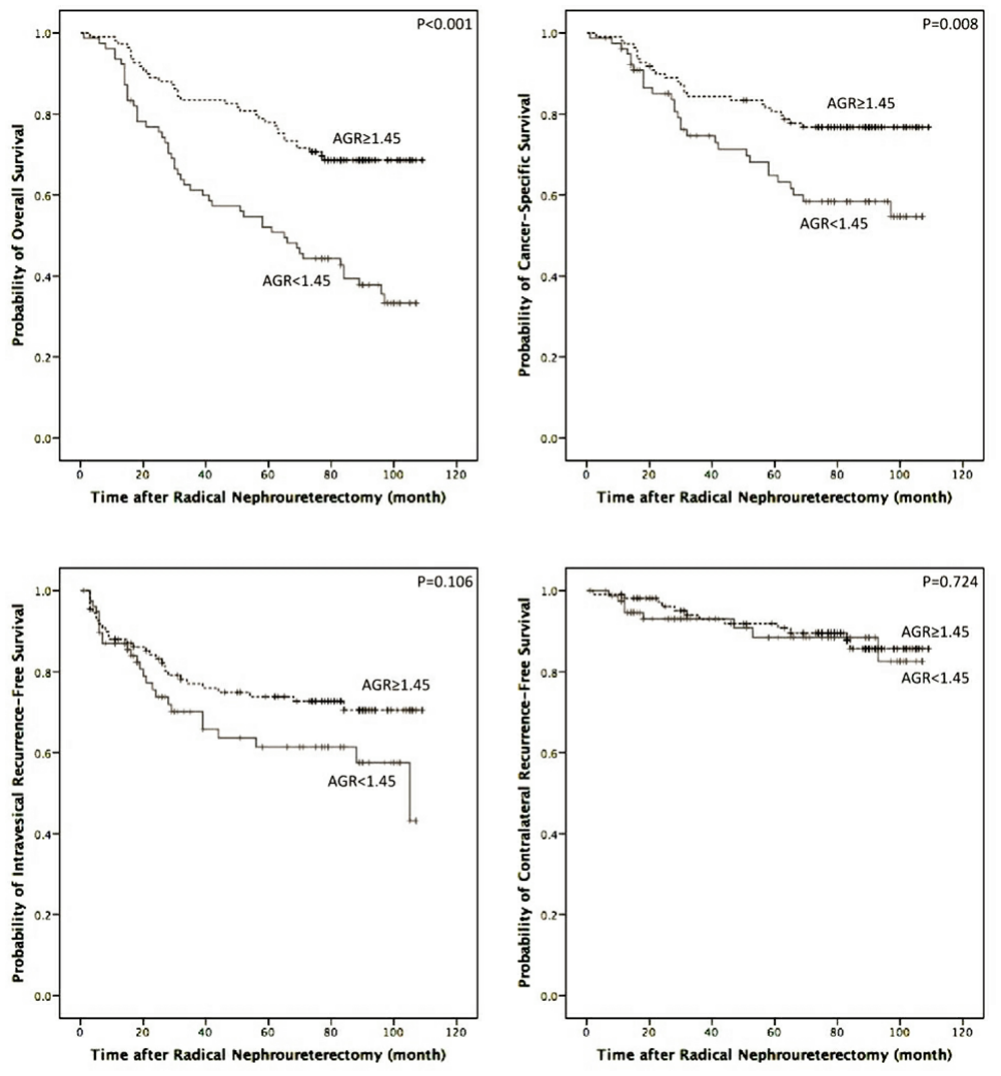
OS, CSS, IRFS and CRFS curve of patients with UTUC after radical nephroureterectomy (AGR≥1.45 vs. <1.45).

During follow-up, 55 patients (29.4%) experienced intravesical recurrence, 20 (10.7%) experienced contralateral recurrence, 83 (44.4%) died and 54 (28.9%) suffered from cancer-specific death in this study. The 5-year OS, CSS, IRFS and CRFS rates were 67.2±3.4%, 74.6±3.3%, 69.4±3.4% and 90.6±2.3%%, respectively. The patients with an AGR < 1.45 had significantly poorer OS and CSS (*P* < 0.001 and *P* = 0.008) than those with an AGR ≥ 1.45 in the Kaplan-Meier survival analysis (Fig 3). For the patients with an AGR < 1.45 and ≥ 1.45, the 5-year OS rates were 37.8±5.7% and 78.0±4.0% and the 5-year CSS rates were 64.8±5.8% and 80.6±3.8%, respectively. Nevertheless, there were no significant differences in IRFS (*P* = 0.106) or CRFS (P = 0.724) between the two groups (Fig 3).

The univariate analysis revealed that preoperative AGR was a statistically significant indicator of OS and CSS; therefore, a multivariate analysis for OS and CSS was performed to determine various prognostic indicators (Table 2). The following factors were included in the multivariate Cox proportional hazards model: age (≥70 vs. <70 years), gender (male vs. female), preoperative CKD stage (No CKD/Stage1/Stage2 as reference), hydronephrosis (present vs. absent), ureter involvement (present vs. absent), tumor architecture (papillary vs. sessile), T stage (advanced vs. localized), lymph node status (N_+_ vs. N_0_/N_x_), tumor grade (high vs. low), and AGR (≥1.45 vs. <1.45). The results showed that AGR was an independent prognostic indicator of both OS and CSS (*P* = 0.002 and *P* = 0.015, respectively). Gender and pathological T stage were also identified as independent prognostic indicators of OS and CSS. Furthermore, age and preoperative CKD stage were independent predictive factors of OS, and ureter involvement was a statistically significant indicator of CSS.

**Table 2:** Cox proportional hazard univariate and multivariate analysis predicting OS and CSS in 187 patients with UTUC.

**OS**	**Univariate analysis**	**Multivariate analysis**
	**HR (95% CI), P value**	**HR (95% CI), P value**
Age, year (≥70 vs. <70)	1.888 (1.199–2.971), 0.006	1.742 (1.099–2.762), 0.018
Gender (male vs. female)	1.900 (1.230–2.934), 0.004	2.745 (1.723–4.374), <0.001
Body mass index, kg/m^2^ (continuous)	0.969 (0.912–1.030), 0.309	
Preoperative CKD stage		
No CKD/Stage 1/Stage 2 (reference)	1	1
Stage 3	1.948 (1.168–3.248), 0.011	1.536 (0.884–2.668), 0.128
Stage 4/Stage 5	2.322 (1.264–4.267), 0.007	2.414 (1.259–4.630), 0.008
Cigarette smoking (yes vs. no)	1.259 (0.709–2.237), 0.432	
Previous or synchronous BUC (yes vs. no)	1.197 (0.684–2.094), 0.529	
ASA score (III vs. ≤II)	1.425 (0.885–2.292), 0.145	
Hydronephrosis (present vs. absent)	1.368 (0.889–2.105), 0.154	
Surgical procedure (laparoscopic vs. open)	0.732 (0.450–1.193), 0.211	
Ureter involvement (present vs. absent)	1.702 (1.103–2.627), 0.016	1.554 (0.986–2.450), 0.058
Multifocality (yes vs. no)	0.770 (0.457–1.298), 0.327	
Tumor architecture (papillary vs. sessile)	0.599 (0.347–1.034), 0.066	
T stage (advanced vs. localized)	2.868 (1.813–4.537), <0.001	2.442 (1.502–3.970), <0.001
Lymph node status (N_+_ vs. N_0_/N_x_)	2.326 (0.940–5.759), 0.068	
Tumor grade (high vs. low)	2.756 (1.422–5.341), 0.003	1.793 (0.975–3.296), 0.060
Lymphovascular invasion (present vs. absent)	1.015 (0.551–1.872), 0.961	
NLR (continuous)	1.151 (0.986–1.343), 0.075	
AGR (≥1.45 vs. <1.45)	0.391 (0.252–0.606), <0.001	0.452 (0.271–0.751), 0.002
**CSS**	**Univariate analysis**	**Multivariate analysis**
	**HR (95% CI), P value**	**HR (95%CI), P value**
Age, year (≥70 vs. <70)	1.649 (0.947–2.869), 0.077	
Gender (male vs. female)	2.868 (1.628–5.052), <0.001	4.257 (2.304–7.864), <0.001
Body mass index, kg/m^2^ (continuous)	0.979 (0.909–1.055), 0.585	
Preoperative CKD stage		
No CKD/Stage 1/Stage 2 (reference)	1	
Stage 3	1.757 (0.961–3.213), 0.067	
Stage 4/Stage 5	1.437 (0.640–3.226), 0.380	
Cigarette smoking (yes vs. no)	1.596 (0.823–3.097), 0.167	
Previous or synchronous BUC (yes vs. no)	1.072 (0.524–2.194), 0.849	
ASA score (III vs. ≤II)	1.422 (0.792–2.551), 0.238	
Hydronephrosis (present vs. absent)	1.944 (1.133–3.335), 0.016	1.233 (0.688–2.211), 0.481
Surgical procedure (laparoscopic vs. open)	0.713 (0.388–1.311), 0.277	
Ureter involvement (present vs. absent)	2.688 (1.525–4.737), 0.001	2.470 (1.314–4.641), 0.005
Multifocality (yes vs. no)	0.712 (0.367–1.380), 0.314	
Tumor architecture (papillary vs. sessile)	0.451 (0.242–0.843), 0.013	1.098 (0.550–2.193), 0.790
T stage (advanced vs. localized)	3.298 (1.889–5.757), <0.001	2.050 (1.104–3.805), 0.023
Lymph node status (N_+_ vs. N_0_/N_x_)	3.728 (1.481–9.389), 0.005	2.017 (0.738–5.510), 0.171
Tumor grade (high vs. low)	3.000 (1.283–7.016), 0.011	2.428 (0.955–6.173), 0.063
Lymphovascular invasion (present vs. absent)	1.057 (0.499–2.241), 0.884	
NLR (continuous)	1.165 (0.964–1.408), 0.113	
AGR (≥1.45 vs. <1.45)	0.490 (0.287–0.838), 0.009	0.474 (0.261–0.864), 0.015

OS, overall survival; CSS, cancer-specific survival; UTUC, upper tract urothelial carcinoma; CKD, chronic kidney disease; BUC, bladder urothelial carcinoma; ASA, American Society of Anesthesiologists; NLR, neutrophil-lymphocyte ratio; AGR, albumin-globulin ratio.

**Fig 4:**
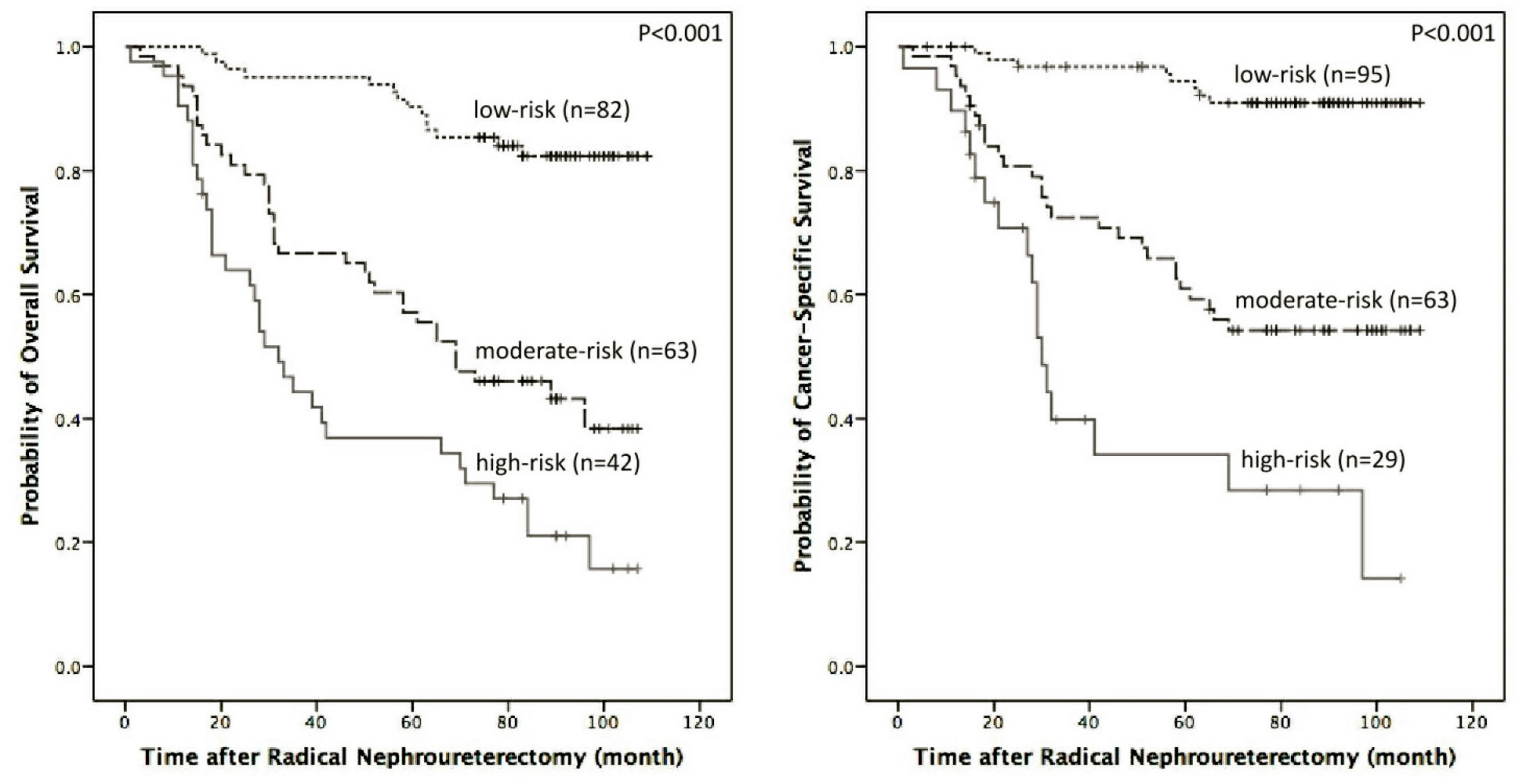
OS and CSS stratified by independent risk factors by multivariate analysis (low-risk: no or 1 independent risk factor; moderate-risk: 2 independent risk factors; high-risk: 3 or more independent risk factors).

Finally, patients with no or one independent risk factor were classified as the low-risk group, patients with two independent risk factors were classified as the moderate-risk group and patients with three or more independent risk factors were classified as the high-risk group. The results of the Kaplan–Meier survival analysis for OS and CSS with risk stratification are illustrated in Fig 4.

## Discussion

Our results showed that preoperative AGR is significantly associated with OS and CSS in patients with UTUC. After adjusting for cofounding factors in the Cox proportional hazard multivariable analysis, the independent predictive value of AGR remained. Patients with low preoperative AGR (<1.45) suffered a poorer OS and CSS than those in the high AGR group (≥1.45), although IRFS and CRFS were comparable between the two groups. Previous studies have evaluated the prognostic value of preoperative AGR in several malignancies (Table 3). To our knowledge, this is the first study to demonstrate the prognostic significance of preoperative AGR in patients with UTUC.

**Table 3:** Previous studies evaluating the predictive value of AGR in other cancers.

**Reference**	**Cohort**	**Grouping method/AGR threshold**	**Findings**
Azab et al. [11]	651 patients with colorectal cancer	Divided into 3 equal tertiles according to the 33rd and 66th AGR percentile.	AGR<1.028 had a significant higher 4-year mortality compared to AGR 1.028–1.321 and AGR>1.321 (42 vs. 19 and 7%, *P*<0.0001).
Azab et al. [12]	354 patients with breast cancer	Divided into 3 equal tertiles according to the 33rd and 66th AGR percentile.	AGR>1.45 predicted a lower 5-year mortality rate compared with AGR 1.21–1.45 and AGR<1.2 (6% vs. 18% and 32%, *P*<0.001).
Du et al. [13]	694 patients with nasopharyngeal carcinoma	AGR = 1.4 which best discriminated good and poor distant metastasis-free survival among all possible thresholds tested	AGR <1.4 was an independent predictor of poor OS (*P* = 0.029) and distant metastasis-free survival (*P* = 0.033) on multivariate analysis.
Duran et al. [14]	240 patients with lung adenocarcinoma	Divided into 3 equal tertiles according to the AGR values.	The high and moderate AGR tertiles showed higher survival rates than the low AGR tertile (*P*<0.0001).

AGR, albumin-globulin ratio; OS, overall survival.

In the present study, AGR was calculated as AGR = albumin/(total protein—albumin), and it reflected both malnutrition and systemic inflammation in UTUC patients. As a major element of serum total protein, albumin may affect cancer through several mechanisms. Albumin has an antioxidant effect against carcinogens (e.g., nitrosamine and aflatoxin), stabilizes cell growth and DNA replication, and buffers sex hormone homeostasis to prevent sex hormone-induced malignancy [17]. Laursen I et al [18] reported that albumin inhibits the proliferation of human breast cancer cell lines by modulating the activities of autocrine growth regulatory factors in vitro. On the other hand, malnutrition and systematic inflammation have been shown to suppress albumin synthesis, even leading to hypoalbuminemia (<35 g/L) [19, 20]. Malnutrition, which is reflected by reduced albumin, weakened several human immune defense mechanisms, which decreased the response to treatment in cancer patients [21, 22]. Recently, several studies demonstrated that hypoalbuminemia was more attributed to systematic inflammation than malnutrition in dialysis patients [23, 24]. In the process of cancer-related systemic inflammation, tumor cells and immune cells release a variety of cytokines and growth factors (e.g., interleukin-1β, interleukin-6 and tumor necrosis factor), which modulate the production of albumin and also facilitate tumor progression, subversion of the host immune response, and resistance to cytotoxic drugs [9, 17, 25–28]. Serum albumin has been used to evaluate prognosis of numerous cancers, including UTUC, and the results of those studies have been fairly consistent: lower serum albumin is an independent predictor of poorer clinical outcome [10, 27]. Non-albumins (total protein—albumin) consist of various proinflammatory proteins, including CRP, complement components and immunoglobulins. Previous studies have shown that an increased preoperative CRP level predicts a poor survival in patients with UTUC [29, 30]. Furthermore, high alpha and gamma globulin levels were shown to be associated with poor survival in lung cancer patients, and high complement 3 and IgA levels were shown to predict poor prognosis in patients with colorectal cancer [31, 32].

Preoperative systemic inflammation has been reported to be associated with poor survival in several cancer types [33, 34]. Many preoperative parameters of systemic inflammation, including CRP, white blood cell count, neutrophil count, neutrophil-lymphocyte ratio and erythrocyte sedimentation rate, have been shown to predict survival in patients with UTUC [30, 35–37]. AA, which has been shown to be highly associated with both CKD and UTUC in China, was reported to induce an inflammatory response and to increase the expression of various proinflammatory genes, including interleukin-1β and tumor necrosis factor, in zebrafish embryos [38, 39]. CKD, which is a common complication in Chinese UTUC patients, has been shown to be associated with a complex state of immune dysfunction that results in chronic inflammation. Additionally, the correlation analysis in our study showed that AGR was negatively correlated with leukocyte, neutrophil and platelet counts, making it a reliable parameter to reflect inflammation, which was consistent with previous studies on the prognostic value of AGR in several other cancers [11–13]. All of these findings lead us to conclude that systemic inflammation plays a vital role in patients with UTUC and that this enhances the predictive value of AGR, especially in the Chinese population.

We believe that both malnutrition and systemic inflammation negatively affect the oncological outcome of patients with UTUC. AGR, a combinatorial measure of serum albumin and non-albumins, reflects the two above-mentioned adverse states, which makes it a more powerful prognostic predictor than albumin alone. In addition, AGR is not affected by dehydration or fluid retention, whereas albumin concentrations vary with these physiologic and pathologic conditions. Therefore, we believe that AGR is superior to serum albumin as a prognostic indicator with low cost, easy application and broad availability. In addition, AGR can be used in the establishment of a pretreatment risk stratification model in the future, which will assist clinicians in selecting UTUC patients who are candidates for neo-adjuvant therapy.

In the present study, an ideal cutoff value for AGR of 1.45 was determined according to the ROC analysis, which was close to the ideal cutoff value reported in a previous study on nasopharyngeal carcinoma (1.4) [13]. The use of different AGR cutoff values in a particular cohort of UTUC patients may lead to different results of the survival comparisons; therefore, an optimal and generalizable AGR threshold for UTUC needs to be determined.

This study was limited by the inclusion of a single center, the short follow-up duration, and its retrospective design. In addition, the levels of many specific inflammatory markers, such as CRP, interleukin and tumor necrosis factor, were unavailable, and therefore, their association with AGR and oncological outcome in patients with UTUC could not be examined. However, within the limitations of an early-phase study for biomarker assessment, our findings suggest that AGR is a potential prognostic indicator of long-term survival in UTUC. Further analyses of larger UTUC cohorts are needed to evaluate the value of AGR as a prognostic marker in UTUC and may serve to improve current risk stratification methods and treatment outcomes in this group of patients.

In conclusion, low AGR was found to be associated with female sex, high preoperative CKD stage and tumor grade in patients with UTUC. In addition, AGR was found to be an independent significant predictor of both OS and CSS in this cohort. These findings suggest that preoperative AGR is a promising blood-based biomarker that will improve the prediction of UTUC outcomes and assist clinicians in therapeutic decision-making. We raised two hypotheses regarding the potential association between AGR and AA exposure in UTUC patients. Further prospective studies are warranted to validate our findings.
